# Identification of the Prognostic Value of Immune-Related Genes in Esophageal Cancer

**DOI:** 10.3389/fgene.2020.00989

**Published:** 2020-08-21

**Authors:** Xiong Guo, Yujun Wang, Han Zhang, Chuan Qin, Anqi Cheng, Jianjun Liu, Xinglong Dai, Ziwei Wang

**Affiliations:** ^1^Department of Gastrointestinal Surgery, The First Affiliated Hospital of Chongqing Medical University, Chongqing, China; ^2^Department of Pathology, Daping Hospital, Army Military Medical University, Chongqing, China; ^3^Department of Digestive Oncology, Three Gorges Hospital, Chongqing University, Chongqing, China; ^4^Department of Gastrointestinal Surgery, Three Gorges Hospital, Chongqing University, Chongqing, China

**Keywords:** esophageal cancer, immune-related gene, TCGA, prognostic model, bioinformatics analysis

## Abstract

Esophageal cancer (EC) is a serious malignant tumor, both in terms of mortality and prognosis, and immune-related genes (IRGs) are key contributors to its development. In recent years, immunotherapy for tumors has been widely studied, but a practical prognostic model based on immune-related genes (IRGs) in EC has not been established and reported. This study aimed to develop an immunogenomic risk score for predicting survival outcomes among EC patients. In this study, we downloaded the transcriptome profiling data and matched clinical data of EC patients from The Cancer Genome Atlas (TCGA) database and found 4,094 differentially expressed genes (DEGs) between EC and normal esophageal tissue (*p* < 0.05 and fold change >2). Then, the intersection of DEGs and the immune genes in the “ImmPort” database resulted in 303 differentially expressed immune-related genes (DEIRGs). Next, through univariate Cox regression analysis of DEIRGs, we obtained 17 immune genes related to prognosis. We detected nine optimal survival-associated IRGs (*HSPA6, CACYBP, DKK1, EGF, FGF19, GAST, OSM, ANGPTL3, NR2F2*) by using Lasso regression and multivariate Cox regression analyses. Finally, we used those survival-associated IRGs to construct a risk model to predict the prognosis of EC patients. This model could accurately predict overall survival in EC and could be used as a classifier for the evaluation of low-risk and high-risk groups. In conclusion, we identified a practical and robust nine-gene prognostic model based on immune gene dataset. These genes may provide valuable biomarkers and prognostic predictors for EC patients and could be further studied to help understand the mechanism of EC occurrence and development.

## Introduction

Esophageal cancer (EC) is ranked 7th and 6th in incidence and mortality, respectively (Bray et al., 2018). It is one of the most aggressive types of cancer. Although the addition of neoadjuvant or perioperative therapy provides a modest improvement in overall survival in resectable cases, the prognosis of patients with advanced EC is still very poor (Cunningham et al., 2006; Allum et al., 2009; van Hagen et al., 2012; Noble et al., 2017). Due to recurrence, extensive invasion and metastasis, the overall 5-year survival rate of EC is lower than 13% after initial diagnosis (Khalil et al., 2016; Vo et al., 2019). Hence, identifying biomarkers for the treatment and prognostic prediction of EC could lead to better interventions for patients with an otherwise poor prognosis.

Immune disorders in tumor is regarded as a promoting factor during tumorigenesis and development. In recent years, immunotherapy has become a promising potential therapy for various cancers in addition to surgery and radiotherapy (Khalil et al., 2016; Zhao et al., 2019). EC cells harbor abundant tumor antigens, including tumor-associated antigens and neoantigens, which have the ability to initiate dendritic cell-mediated tumor-killing cytotoxic T lymphocytes in the early stage of cancer development. As EC cells battle the immune system, they obtain an ability to suppress antitumor immunity through immune checkpoints, secreted factors, and negative regulatory immune cells (Huang and Fu, 2019). Immune checkpoint inhibitors (ICIs) have been investigated in various types of cancers and provide a new treatment landscape (Tanaka et al., 2017). ICIs have been reported to attenuate tumor growth mainly by reducing the immune escape of cancer cells, and programed death 1 (*PDL1*) is one of the immune checkpoints that is the most commonly used target for immunotherapy in EC (Shaib et al., 2016). However, at present, EC immunotherapies always lead to mixed results, which are partially caused by the absence of reliable markers that are predictive of treatment response (Ohashi et al., 2015). Molecular profiles of tumor cells and cancer-related cells within their microenvironments represent promising candidates for predictive and prognostic biomarkers. Despite vigorous efforts have been made with major breakthroughs in high-throughput genomic technologies (Li et al., 2017). Increasing evidence suggests that the expression of IRGs may be related to the prognosis of tumors. Qiu et al. (2020) identified and verified of an individualized prognostic signature of bladder cancer based on seven immune related genes. Zhang et al. (2020) discovered a novel immune-related gene signature for risk stratification and prognosis of survival in lower-grade glioma. And Zhao et al. (2020) used immune score to predict survival in early-stage lung adenocarcinoma patients.

Similarly, the prognostic characteristics based on these IRGs may help in the diagnosis and individualized treatments for EC (Gentles et al., 2015). However, several studies have reported the relationship of IRGs with the prognosis of patients with EC (Turato et al., 2019; Yan et al., 2019). In addition, there is currently no systematic description or study of IRGs and the tumor immune microenvironment in large samples of patients with EC. Therefore, a systematic description and analysis of the tumor immune microenvironment and IRGs impact on prognosis is necessary for EC immunotherapy and patient prognosis. In this study, we analyzed 182 samples of EC in the TCGA database, and 303 differentially expressed IRGs were found. Through multivariate Cox regression analysis, we found 9 immune-related prognosis genes. An accurate model for evaluating the prognosis of patients was established, and we investigated the clinical utility of this model in patients with EC. In addition, we calculated the correlation between immune cell infiltration and risk score in the tumor microenvironment. Our study identified new biomarkers and prognostic factors for EC, thus provides some new therapeutic targets in EC.

## Materials and Methods

### Data Acquisition and Processing

The RNA-Seq gene expression profiles of patients with EC, including the Fragments Per Kilobase of transcript per Million Mapped reads (FPKM) based on the Illumina (San Diego, CA, United States) HiSeq 2000 RNA sequencing platform, were downloaded from the TCGA database using the GDC-client download tool^1^ (Cao et al., 2019). The workflow type is HTSeq-FPKM. Then, the “limma” package of R software was utilized for the normalization of RNA expression profiles and averaged the duplicate data to remove the batch effects. Clinical data for the corresponding EC patients were also retrieved from the TCGA database, which included gender, age, tumor stage, and survival information. The patient’s TCGA ID was used to distinguish between a tumor sample and a normal sample. The detailed characteristics and histopathological features of the EC patients and their TCGA IDs are summarized in Supplementary Table S1.

Immunologically relevant list of genes curated with functions and Gene Ontology terms (immune-related gene list) were download from the resources section of the “ImmPort” database^2^ (Bhattacharya et al., 2018). It contains a total of 2,496 genes defined as immune-related. Data regarding 318 cancer-associated transcription factors (TFs) were obtained from the “Cistrome” project^3^ (Mei et al., 2017).

### Criteria of Enrolled Patients for the Construction of Risk Signature

The inclusive criteria of patients with EC for model construction were as follows: (1) patients primarily diagnosed with EC, (2) with only adenocarcinoma or squamous cell carcinoma as pathological type, (3) only samples with RNA-sequencing data, (4) patients with complete clinicopathological parameters, (5) overall survival time is more than 30 days.

### Identification of Differentially Expressed Genes, Differentially Expressed IRGs

Differentially expressed genes (DEGs) between EC and normal tissues were identified using Wilcoxon test after within-array replicate probes were replaced with their average via “limma” package in the R software (version 3.6.2). | Log_2_ fold change (FC)| >2.0 and false discovery rate (FDR) adjusted to less than 0.05 were set as the cutoff criteria. Then, the DEGs were intersected with the immune-related gene list to obtain the DEIRGs. Those significant DEGs are visualized using heatmaps and volcano plots via “pheatmap” package in the R software. In addition, an online database, GEPIA 2.0 (Tang et al., 2019), was used to analyze differential expression of prognostic genes between 286 GTEx normal samples and 182 TCGA tumor samples.

### Functional Annotations and Signaling Pathway Enrichment Analysis

“Clusterprofiler” R package (Yu et al., 2012) was used for Gene Ontology (GO) annotation and Kyoto Encyclopedia of Genes and Genomes (KEGG) pathway enrichment analysis of DEGs and IRGs. The results of GO annotation and KEGG pathway analyses were visualized using the “GOplot” package in R platform. Gene Set Enrichment Analysis (GSEA) software (version 4.0.1) was used to analyze pathway activation and inhibition in high-risk and low-risk patients.

### Risk Score Calculation and Survival Analysis

To explore candidate prognostic biomarkers of EC, a joint cox regression analysis was performed. Firstly, we merged the expression levels of IRGs with the corresponding survival time and survival status data of EC patients. Then, a univariate Cox proportional hazard regression analysis was used to identify the candidate survival-associated IRGs when *p*-value < 0.05. Next, the least absolute shrinkage and selection operator (LASSO) Cox regression analysis was used to identify the genetic model with the best prognostic value by using “glmnet” package in R software. Finally, multivariate Cox regression analysis was employed to construct the prognosis signature for predicting the prognosis in EC patients. We calculated the risk score of each patient using the expression of DEIRGs and the regression coefficients obtained in the regression model. The coefficient of the gene is multiplied by the expression of the gene and then summed to obtain each patient’s risk score. The calculation formula is below (Wan et al., 2019):

(1) $$R\,i\,s\,k\,s\,c\,o\,r\,e\,\left(p\,a\,t\,i\,e\,n\,t\,s\right)=\sum_{i=1}^{n}\text{coefficien}\,t\,\left({\text{gene}}_{i}\right)\,\text{expression}\mathrm{value}\,\mathrm{of}\,\left({\text{gene}}_{i}\right)\mathrm{value}\,\mathrm{of}\,\left({\text{gene}}_{i}\right)$$

Here, “gene_i_” is the ith selected gene, and “coefficient (gene_i_)” is the estimated regression coefficient of gene_i_ from the Cox proportional hazards regression analysis. Time-dependent receiver operating characteristic (ROC) curves were used to assess the accuracy of prognostic prediction models. The area under the ROC curve (AUC) >0.60 was considered an acceptable prediction, and an AUC >0.75 was recognized as an excellent predictive value. For survival analysis, patients were divided into low- and high-risk groups according to the median risk score calculated by this prognostic model, and then log-rank tests were used to analyze the survival data.

### Construction of Cancer-Associated TFs and IRG Regulatory Networks

Differentially expressed transcription factors (DETFs) were derived from the intersection of tumor-associated TFs and DEGs. DETFs and survival-associated IRGs samples with the same TCGA patient ID were then used for correlation testing. *p* < 0.05 and cor ≥ 0.3 were considered significant correlations. Then, cytoscape software (Shannon et al., 2003) was used to draw the regulatory network.

### Construction of a Predictive Nomogram Based on the IRGs

A nomogram encompassing the risk score based on expression of prognostic IRGs and clinicopathological factors was constructed using the “rms” R package. Based on the different clinicopathological characteristics and the risk score of each patient, we calculated the total score to predict 1, 2, and 3-year prognosis of EC patients.

### Clinical Correlation Analysis

Univariate regression analysis and multivariate regression analysis were used to identify factors (including gender, age, TNM stage and risk score) affecting survival and independent prognostic factors in patients with EC. The correlation between survival-associated IRGs and clinicopathological characteristics was analyzed in R platform. *p* < 0.05 was considered to have a significant correlation.

### Relationship Between Risk Score and Immune Cell Infiltration

The immune cell infiltrate data were collected from Tumor Immune Estimation Resource (TIMER)^4^ (Liu et al., 2011) database. The database includes 10,897 samples across 32 cancer types from TCGA to estimate the abundance of six subtypes of tumor-infiltrating immune cells, including B cells, CD8 T cells, CD4 T cells, dendritic cells (DCs), macrophages, and neutrophils. Based on the same patient’s ID as TCGA, the correlation between patient immune infiltrated cells and risk score was calculated in R software.

### Statistical Analyses

All data were processed with R (version 3.6.2) and Perl (5.30.1) software. DEGs were identified using the Wilcox test. Survival analyses were performed using the Kaplan-Meier method and the log-rank test.

## Results

### Differentially Expressed IRGs in EC

**FIGURE 1:**
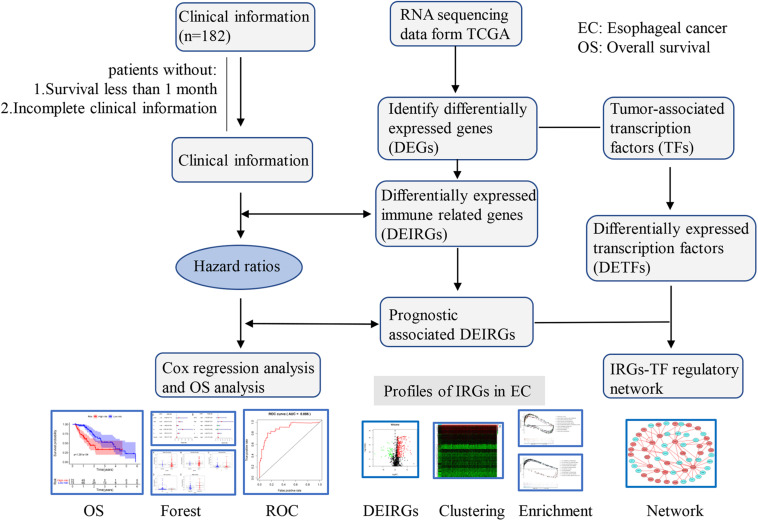
Flowchart of the study. RNA-Seq data and corresponding clinical information of EC cohort were downloaded from the TCGA data portal. After excluding patients with incomplete clinical data and duplications, the complete data was used for subsequent analysis.

**FIGURE 2:**
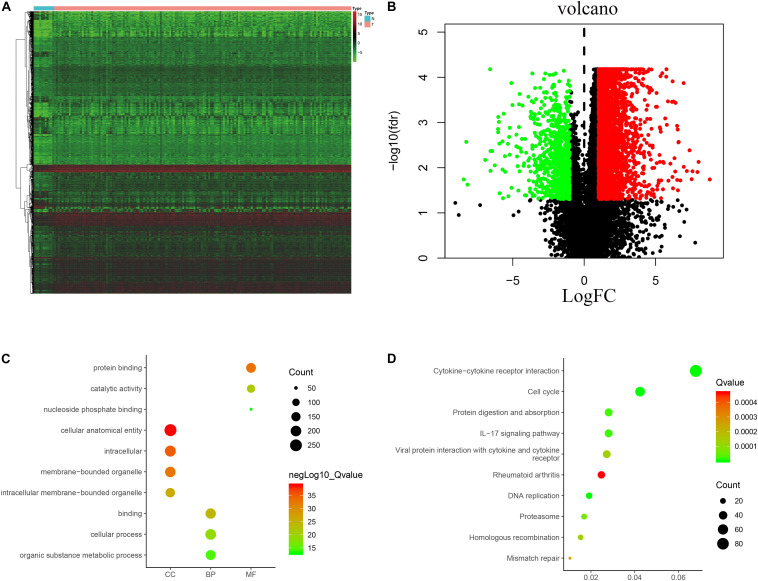
Expression of genes and function enrichment. Heatmap **(A)** and volcano plot **(B)** showing the DEGs between EC and normal esophageal specimen. Red dots represent up-regulated and green dots represent down-regulated DEGs, black dots represent no difference, respectively (fold change >2, *p* < 0.05). GO **(C)** and KEGG **(D)** showing the differentially expressed immune-related genes. **(C)** GO analysis results showing that DEGs were particularly enriched in BP, CC, and MF. **(D)** The significantly enriched pathways of the DEGs determined by KEGG analysis. GO, gene ontology; BP, biological process; CC, cell component; MF, molecular function; KEGG, Kyoto Encyclopedia of Genes and Genomes.

**FIGURE 3:**
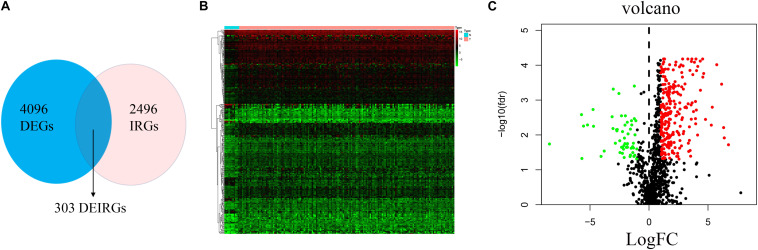
Differential expression of immune-related genes. **(A)** The intersection of DEGs and IRGs. Heatmap **(B)** and volcano plot **(C)** showing the DEGs between EC and normal esophageal specimen. Red dots represent up-regulated and green dots represent down-regulated DEGs, black dots represent no difference, respectively (fold change >2, *p* < 0.05).

The analysis process for this study is shown in Figure 1. A total of 182 patients were involved in the development and validation of the prognostic signature, including 95 squamous cell neoplasms, 87 adenomas and adenocarcinomas. Of these, 111 were white people, 46 were Asian, five were African American, and 20 were unreported. The TCGA IDs for the 182 patients were presented in Supplementary Table S1. Initially, we downloaded and normalized the mRNA expression data of 182 patients with EC from the TCGA database and eliminated partial incomplete data. Then, we performed a differential expression analysis using Wilcoxon test with a log_2_(FC) > 1 and *p* < 0.05. We found 4,094 DEGs between 10 normal samples and 162 tumor samples (Figures 2A,B). The DEGs list, including log_2_FC and the FDR adjusted *p*-values of each gene was provided in Supplementary Table S2. Then, we performed GO and KEGG pathway analysis for the DEGs and the top 10 GO and KEGG pathway enrichment terms shown in Figures 2C,D. The KEGG analysis indicated that the genes were mainly involved in cytokine-cytokine receptor interaction and cell cycle signaling pathway, which are pivotal in the regulation of immune responses (Murphy and Murphy, 2010; Zhang J. et al., 2018). Next, we downloaded the list of IRGs from the “ImmPort” database. These IRGs intersect with the DEGs, and 303 differentially expressed IRGs were obtained (Figure 3A), including 56 down-regulated and 247 up-regulated genes (Figures 3B,C).

### Prognostic Immune Signatures in EC

**FIGURE 4:**
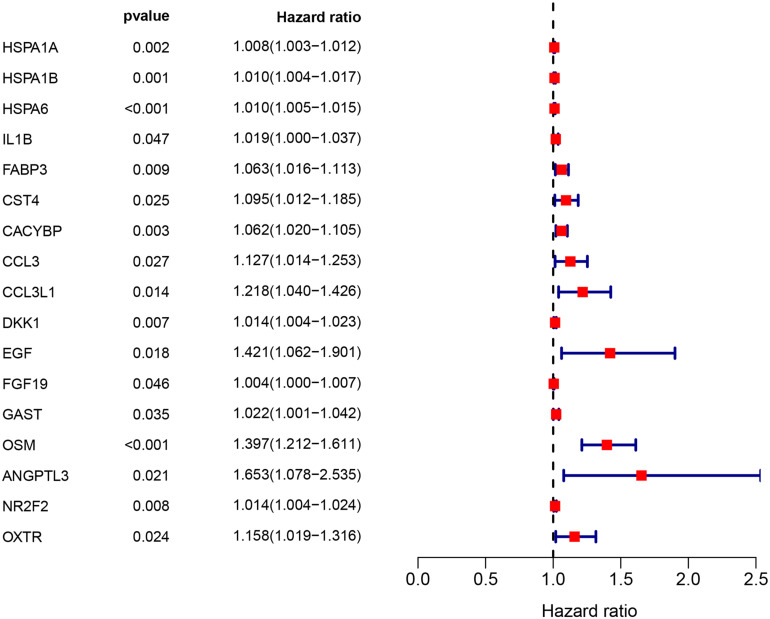
The prognostic value of prognostic associated IRGs in EC. Univariate regression analysis of IRGs related to survival. *p* < 0.05 indicates a significant correlation between genes and prognosis, hazard ratio (HR) value >1 means that the gene is a high-risk gene, and HR <1 means a low-risk gene.

Clinical EC data corresponding to RNA sequencing data were downloaded from the TCGA database, and data with a survival time of less than 1 month were excluded. Then, we merged the survival time and survival status of each patient with gene expression data. Then, we set filter criteria of *p* < 0.05 and used univariate Cox regression analysis. Seventeen (*HSPA1A, HSPA1B, HSPA6, IL1B, FABP3, CST4, CACYBP, CCL3, CCL3L1, DKK1, EGF, FGF19, GAST, OSM, ANGPTL3, NR2F2*, and *OXTR*) prognostic immune signatures were obtained (Figure 4).

### Establishment and Verification of Prognostic Model

**FIGURE 5:**
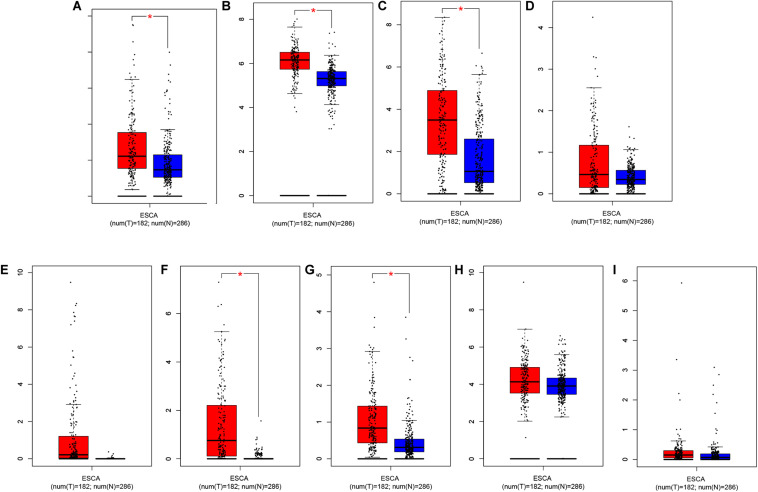
Relative expression of prognostic-related IRGs between EC sample in TCGA database (*n* = 182) and normal esophageal sample form GTEx database (*n* = 286). **(A)** *HSPA6*, **(B)** *CACYBP*, **(C)** *DKK1*, **(D)** *EGF*, **(E)** *FGF19*, **(F)** *GAST*, **(G)** *OSM*, **(H)** *ANGPTL3*, and **(I)** *NR2F2* *p < 0.05.

Through further analysis via Lasso and multivariate Cox proportional hazards regression analysis, we ultimately obtained 9 optimal prognostic immune genes and incorporated them into the prognostic risk model: *HSPA6, CACYBP, DKK1, EGF, FGF19, GAST, OSM, ANGPTL3*, and *NR2F2*. All the 9 genes are high-risk genes, as shown in Table 1. We used gene mRNA levels and risk estimate regression coefficients to calculate risk score for each patient to explore the significance of prognostic genes. The calculation formula is described in the methods. Risk score = (-0.008235 × expression of *HSPA6*) + (0.492 × expression of *CACYBP*)+ (0.014939 × expression of *DKK1*) + (0.29151 × expression of *EGF*) +(0.004 × expression of *FGF19*) + (0.03515 × expression of *GAST*) + (0.327446 × expression of *OSM*) + (0.732285 × expression of *ANGPTL3*) + (0.018484 × expression of *NR2F2*). Then, those prognostic genes were verified between 182 tumor samples of TCGA database and matched 286 normal samples from GETx database (Figure 5). Thus, we found *HSPA6, CACYBP, DKK1, GAST, OSM* were up-regulated in EC tissues (*p* < 0.05 and logFC > 1).

**TABLE 1:** Coefficients and multivariable Cox model results for immune related genes in esophageal cancer.

Gene symbol	Coef	HR	(95%CI)	*p*-value
*HSPA6*	0.008235	1.008269	(1.001731–014852)	0.013119
*CACYBP*	0.043103	1.044046	(0.99238–1.098401)	0.095996
*DKK1*	0.014939	1.015051	(1.004806–1.025401)	0.003942
*EGF*	0.291513	1.338447	(0.993541–1.803087)	0.055194
*FGF19*	0.004144	1.004148	(1.000211–1.008102)	0.038915
*GAST*	0.034152	1.03474	(1.013293–1.05664)	0.001395
*OSM*	0.327446	1.387419	(1.178695–1.633105)	8.27E-05
*ANGPTL3*	0.732285	2.079828	(1.319571–3.278099)	0.001607
*NR2F2*	0.018484	1.018656	(1.00547–1.032014)	0.005427

*coef, coefficient; HR, hazard ratio; CI, confidence interval.*

**FIGURE 6:**
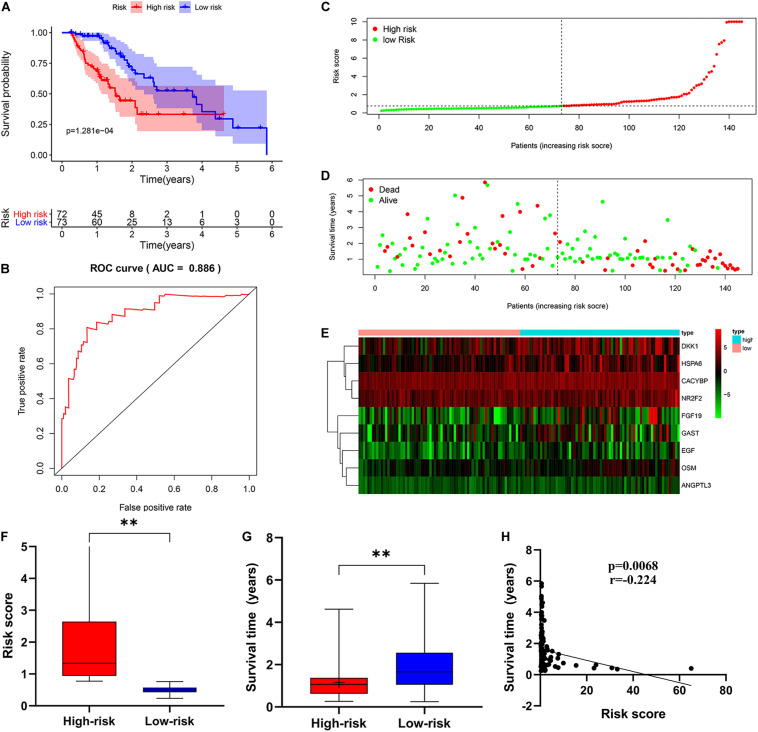
The prognostic value of the immune-related risk score. **(A)** Patients in high-risk group suffered shorter OS. The blue represents the overall survival of patients in the low-risk group; the red represents the overall survival of patients in the low-risk group. **(B)** Survival-dependent receiver operating characteristic (ROC) curve validation of prognostic value of the prognostic index. **(C)** The risk score distribution. Green dots represent risk score for low-risk patients; red dots represent risk score for high-risk patients. **(D)** The relationship between survival status and risk score. The abscissa represents the number of patients, and the ordinate is the risk score. Red dots represent dead patients, green dots are living patients. **(E)** Risk gene expression and risk score (abscissa) in EC patients. **(F)** Risk score in high and low-risk group. **(G)** Patient survival time in high and low-risk group. **(H)** The correlation between survival time and risk score. *p < 0.05, **p < 0.001.

Then, patients were divided into a low-risk group and a high-risk group according to the median risk score. We used the log-rank test to plot survival curves to evaluate the difference in OS between the two groups. As shown in Figure 6A, the prognosis of the low-risk group was significantly better than that of the high-risk group (*p* = 1.281e-04). The 1-year survival rates for the high-risk and low-risk groups were 67% (95% CI: 56.8–79.5%) and 95% (95% CI: 90.14–100%), respectively. The 2-year survival rates for the high-risk and low-risk groups were 38% (95% CI: 25.1–59.9%) and 69% (95% CI: 56.79–84.7%), respectively. Here, because of the poor prognosis in the high-risk group, we could not obtain a complete 5-year survival rate. In order to test the predictive accuracy of the model, we constructed a ROC curve. The AUC value for the prognostic model was 0.886, which illustrates the accuracy of the model (Figure 6B). Then, we ranked patients according to their risk score and analyzed their distribution using the median risk score as the cut-off (Figure 6C). It can be seen that after patients were sorted according to risk score, as the risk score increases, more and more patients die, i.e., the higher the risk score, the greater was the number of deaths. Similarly, the higher the risk score, the shorter the survival time of the patient. The distribution of survival status, survival time and risk score were shown in Figure 6D. As the risk score increases, the expression of high-risk genes also increases, and vice versa. Expression patterns of risk genes in the low-risk group and high-risk group are shown in a heat map (Figure 6E). The risk score in the high-risk group was significantly higher than that in the low risk group (Figure 6F), and the survival time of patients in the high-risk group was significantly lower than that in the low risk group (Figure 6G), and the risk score was negatively correlated with the survival time of patients (Figure 6H). Those results show that the risk score in the model has an accurate predictive effect on the prognosis of patients.

### Independent Prognostic Value of the Risk Model

**FIGURE 7:**
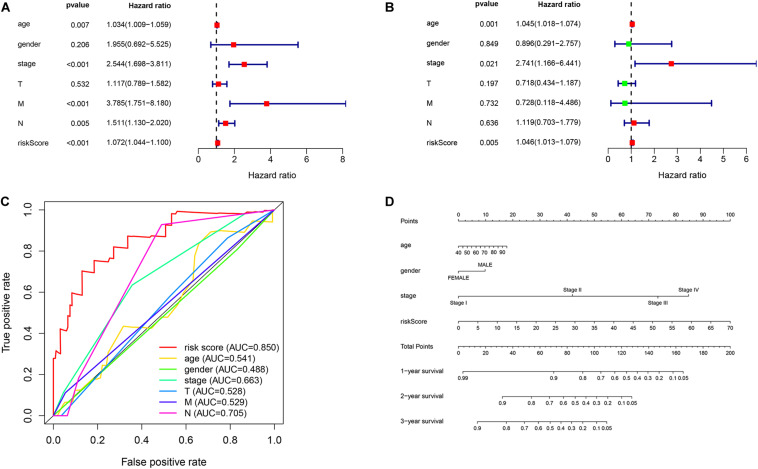
Independent prognostic value of the risk model. **(A)** Univariate and **(B)** multivariate regression analysis of clinical characteristics and risk score as independent prognostic factors. **(C)** The ROC curve evaluated the accuracy of independent prognostic factors for EC. **(D)** A nomogram predict the outcome of EC patients based on their clinical characteristics.

First, we used univariate regression analysis to determine the correlation between clinical characteristics (age, gender, stage, and TNM staging) and prognosis. We found that age (*p* = 0.007), stage (*p* < 0.001), M staging (*p* < 0.001), N staging (*p* = 0.005) and risk score (*p* < 0.001) were significantly correlated with prognosis (Figure 7A). Then, we used multivariate analysis to determine the independent prognostic value of the risk model, and the results showed that age (*p* = 0.001), stage (*p* = 0.021), and risk score (*p* = 0.005) were independently associated with prognosis (Figure 7B). These results indicate that the prognostic risk model can be used to predict the prognosis of patients with EC accurately and independently. Subsequently, we used ROC curves to verify the accuracy of risk score in evaluating prognosis. The fact that the AUC is 0.850 also indicates the exactitude of our model (Figure 7C). Meanwhile, for better prediction of the prognosis of patients with EC at 1, 2, and 3 years after diagnosis, we constructed a new nomogram based on OS-related variables (age, sex, stage, and risk score). The higher the patient’s total score, the worse is their prognosis (Figure 7D).

### Correlation Between the Prognostic Factors and Clinicopathologic Parameters

**FIGURE 8:**
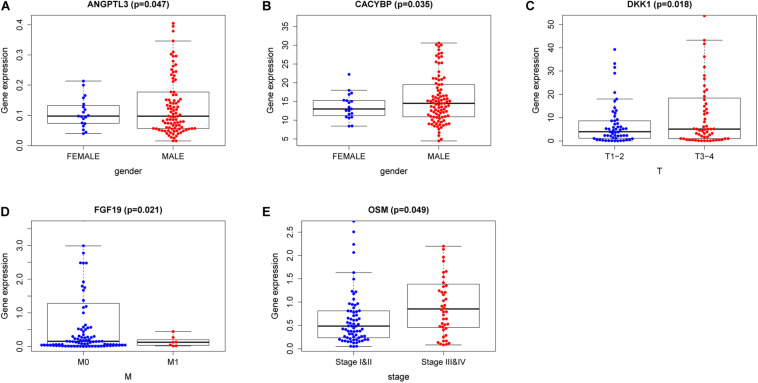
Relationships of the variables in the model with the clinical characteristics of patients. **(A)** *ANGPTL3* expression and gender. **(B)** *CACYBP* expression and gender. **(C)** *DKK1* expression and T staging. **(D)** *FGF19* expression and M stage. **(E)** *OSM* expression and pathological stage. The three horizontal lines in each picture means mean ± SD.

To confirm our model’s ability to predict EC progression, we also analyzed the potential relationship between the risk genes (*HSPA6, CACYBP, DKK1, EGF, FGF19, GAST, OSM, ANGPTL3*, and *NR2F2*), risk score and clinicopathologic parameters, including patient sex, tumor grade, and TNM staging. As shown in Figures 8A,B, *ANGPTL1* and *CACYBP* were significantly overexpressed in female patients. As the expression of DKK1 increases, the risk of T staging increases in patients with EC (Figure 8C). However, as *FGF19* expression decreased, the risk of distant metastasis decreased (Figure 8D). High expression of *OSM* was significantly correlated with high stage (Figure 8E). These results suggest that the development of EC may be related to dysregulated expression of IRGs.

### Immune Cell Infiltration Analysis

**FIGURE 9:**
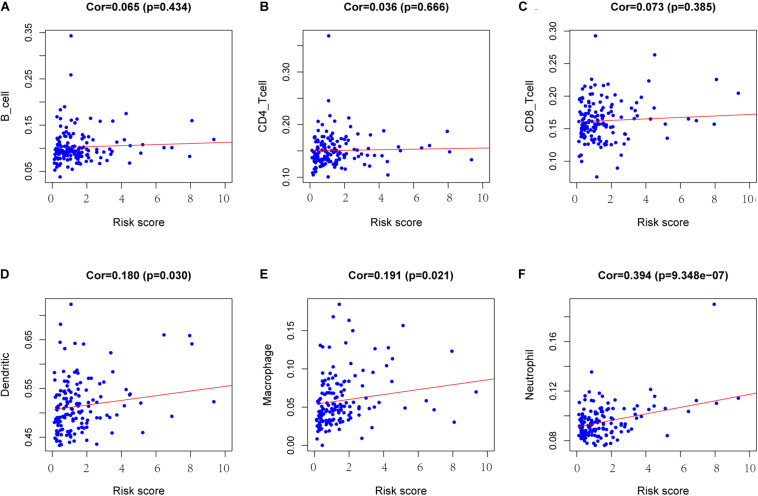
Analysis of the correlation between the risk score and immune cell infiltration. **(A)** B cells. **(B)** CD4+ T cells. **(C)** CD8+ T cells. **(D)** Dendritic cells. **(E)** Macrophages. **(F)** Neutrophils. Cor >0.4 and *p* < 0.05 was used for correlation test.

To determine whether there is a correlation between risk score and tumor infiltration with immune cells (CD8^+^ T cells, CD4^+^ T cells, B cells, macrophages, neutrophils and dendritic cells), we conducted a correlation test between immune cell infiltration and risk score, as shown in Figure 9. The risk score had no significant correlation with B cells (*p* = 0.434), CD4^+^ T cell (*p* = 0.666) or CD8^+^ T cells (*p* = 0.385) (Figures 9A–C). However, the risk score positively correlated with the levels of dendritic cell infiltration (cor = 0.180, *p*-value = 0.030) (Figure 9D), macrophage cells (cor = 0.191, *p*-value = 0.021) (Figure 9E) and neutrophil cells (cor = 0.394, *p*-value = 9.348e-07) (Figure 9F).

### Construction of a Survival-Associated IRG and TF Regulatory Network

**FIGURE 10:**
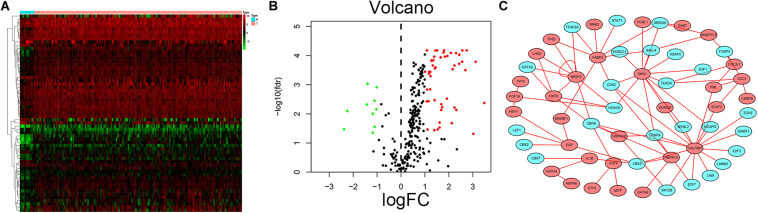
Prognostic associated IRGs and TFs regulatory network. Heatmap **(A)** and volcano plot **(B)** show the differentially expressed transcription factors between EC and esophageal normal specimen. Red dots represent up-regulated and green dots represent down-regulated DEGs, black dots represent no difference, respectively. **(C)** Regulatory network of TFs and prognostic related IRGs; the green nodes represent TFs and the red nodes represent prognostic related IRGs. Correlation coefficient >0.3 and *p* < 0.05.

Transcription factors play an important role in the regulation of genes. To explore possible mechanisms of survival-associated IRG dysregulation in EC, we analyzed the correlation between tumor-related transcription factors (TFs) and survival-associated IRG expression. We screened 60 (FDR < 0.05, log_2_FC > 2) TFs that were differentially expressed between EC and normal tissues from 318 transcription factors in the “Cistrome” database (Figures 10A,B). Next, we used a *p*-value < 0.05 and correlation coefficient >0.3 as the cut-off values to analyze the correlations between the 60 TFs and survival-associated IRGs. Among the 60 TFs, 27 were significantly associated with survival-associated IRGs. To better explain the regulatory relationship, Cytoscape software was used to draw the regulatory network, as shown in Figure 10C.

### Enrichment Analysis of IRGs

**FIGURE 11:**
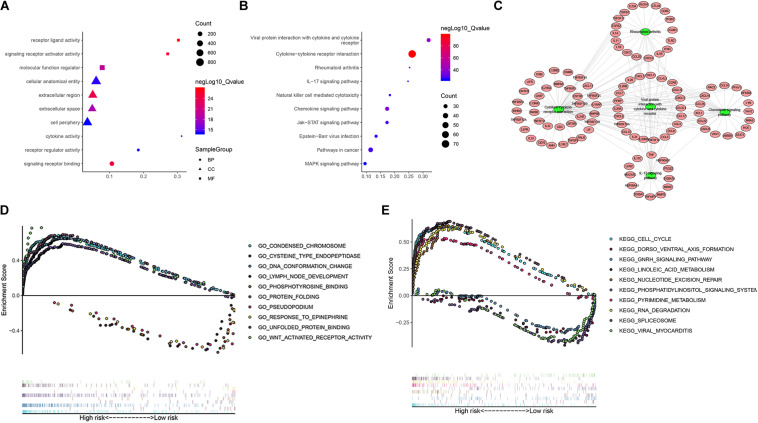
The function enrichment analysis of the IRGs. Differentially expressed IRGs **(A)** Gene Ontology (GO) analysis, **(B)** Kyoto Encyclopedia of Genes and Genomes (KEGG) analysis. **(C)** The networks between IRGs and top 5 enrichment pathway. **(D)** GO analysis of Gene Set Enrichment Analysis (GSEA) in high-risk and low-risk groups, **(E)** KEGG analysis of GSEA in high-risk and low-risk groups.

To further study the potential function and mechanism of IRGs, we performed Kyoto Encyclopedia of Genes and Genomes (KEGG) and Gene Ontology (GO) analysis by using “clusterprofiler” R packages. The top 10 GO enrichment terms included biological process (BP), molecular function (MF) and cell component (CC), as shown in Figure 11A. The KEGG enrichment analysis results show that it is mainly enriched in some key immune-related pathways, such as chemokine signaling pathway, cytokine-cytokine receptor interaction and JAK-STAT signaling pathways (Figure 11B). Based on the relationship between IRGs and KEGG pathways, we constructed a network using Cytoscape to show the genes enriched in the top 5 pathways (Figure 11C). In addition, we also observed which pathways were enriched in patients in the high-risk and low-risk groups by using Gene Set Enrichment Analysis (GSEA) software. The top five GO terms enriched in the high-risk and low-risk groups are shown in Figure 11D, and the top 5 pathways enriched in the high-risk and low-risk groups are shown in Figure 11E. The results showed that key important pathways, such as the cell cycle, pyrimidine metabolism and RNA degradation, were significantly activated in the high-risk group. The GNRH signaling pathway, viral myocarditis, spliceosome pathway and other pathways were active in the low-risk group.

## Discussion

Esophageal cancer (EC) is a clinically challenging disease that requires a multidisciplinary approach (Lagergren et al., 2017). The high fatality rate of EC is a cause of concern around the world. Despite incremental advances in diagnostics and therapeutics, EC still carries a poor prognosis, and thus, there remains a need to elucidate the molecular mechanisms underlying this disease. Increasing evidence shows that a comprehensive understanding of EC requires attention not only to tumor cells but also to the tumor microenvironment (Lin et al., 2016). Further study on the relationship between immune signals and EC occurrence and development will help to develop new and specific targeted therapy strategies, especially in combination therapy, with great potential (Li et al., 2017).

In this study, we performed a comprehensive analysis of IRGs and immune infiltrating cells in EC and linked the data to clinical outcomes and prognosis of patients with EC. First, we systematically studied the IRGs in EC. We identified 303 differentially expressed IRGs. They are mainly enriched in the chemokine signaling pathway, cytokine-cytokine receptor interaction, *NF-*κ*B* signaling pathway and JAK-STAT signaling pathway. Recent research reported that tumor cell-secreted IL-6 and IL-8 impair the activity and function of NK cells via *STAT3* signaling, and contribute to esophageal squamous cell carcinoma malignancy (Wu et al., 2019). *NF-*κ*B* is overexpressed in many solid and liquids tumors, including both ESCC and EAC (Karin et al., 2002). Our results are the same as before, and some of these pathways play an important role in EC (Izzo et al., 2006). Zhang B. et al. (2018) reported on IRGs, specifically that *TSPAN15* interacts with BTRC to promote esophageal squamous cell carcinoma metastasis by activating *NF-*κ*B* signaling and indicated that *TSPAN15* may serve as a new biomarker and/or provide a novel therapeutic target for patients with OSCC. This suggests that IRGs can be used as prognostic biomarkers. To study the underlying mechanisms of EC development, we constructed an IRG-TF regulatory network and found 27 TFs related to prognostic genes; among them, *NR2F2* is both an IRG and TF and is involved in transcriptional regulation.

It makes sense to stratify patients and find predictive prognostic markers. Yuting He et al. found that a new model based on IRGs was effective in predicting prognosis, evaluating disease state, and identifying treatment options for patients with hepatocellular carcinoma (He et al., 2020). Therefore, we used univariate regression analysis to identify IRGs associated with prognosis and tested the value of these survival-associated IRGs for the prognostic stratification of patients. We finally identified the nine best candidate genes (*HSPA6, CACYBP, DKK1, EGF, FGF19, GAST, OSM, ANGPTL3*, and *NR2F2*) through a combination of Cox regression analyses and Lasso regression. These genes were used to construct a Cox regression risk model. This model can predict the outcome of high- and low-risk groups. The accuracy of the model was tested by ROC curve analysis. Then, we found that the risk score could be used as an independent prognostic factor by using univariate and multivariate regression analysis to determine the correlation between clinical characteristics, risk score and prognosis. A nomogram analysis suggested that by combining the clinical characteristics with the risk score, the 1, 2, and 3-year survival rates for EC can be predicted based on the patient’s score.

An increasing number of studies about the tumor microenvironment (TME) have been published in the field of cancer immunotherapy (Fidler, 2003). For example, it has been reported in lung cancer (Shi et al., 2020), endometrial cancer (Chen et al., 2020), cervical squamous cell carcinoma (Pan et al., 2019) and so on. Tumor escape from antitumor immunity is essential for tumor survival and progression. Tumor cells can suppress the antitumor immune response via recruitment of various immune cell populations or expression of inhibitory molecular factors. Therefore, we explored the correlation between risk score and immune infiltrating cells and found that risk score in the model were not correlated with CD8^+^ T cells, B cells, or CD4^+^ T cells but were significantly correlated with dendritic cells, macrophage cells and neutrophil cells. The positive correlation between high risk score and immune cells also confirmed the accuracy of the model.

In conclusion, we constructed a prognostic model of EC based on IRGs that can accurately predict the prognosis of patients with EC. Furthermore, this model may help to identify new therapeutic targets for advanced EC and provide individualized immunotherapy for patients with EC. Further study of these survival-associated IRGs may shed light on the pathogenesis of EC.

## Data Availability Statement

Transcriptomic data and matching clinical data were downloaded from the TCGA GDC portal (https://portal.gdc.cancer.gov/). The 2498 immune genes were obtained from the ImmPort databae (https://www.immport.org/home) (Bhattacharya et al., 2018). Transcription factors (TFs) associated with cancer data and immune cell infiltrate data (including the abundances of CD8+T cells, B cells, macrophages, CD4+T cells, dendritic cells and neutrophils) were both obtained from the Cistrome project (http://www.cistrome.org/) (Liu et al., 2011).

## Author Contributions

XG and YW conceived, designed this research, and assisted in writing the manuscript. HZ, CQ, and XD conducted the data and statistics analysis. JL, AC, and ZW edited and revised the manuscript. ZW was responsible for supervising the study. All authors read and gave final approval of the manuscript.

## Conflict of Interest

The authors declare that the research was conducted in the absence of any commercial or financial relationships that could be construed as a potential conflict of interest.

## Footnotes

1 https://portal.gdc.cancer.gov/

2 https://www.immport.org/home

3 http://www.cistrome.org/

4 http://timer.cistrome.org/
